# Deep Learning for SARS COV-2 Genome Sequences

**DOI:** 10.1109/ACCESS.2021.3073728

**Published:** 2021-04-16

**Authors:** Albert Whata, Charles Chimedza

**Affiliations:** 1 School of Natural and Applied SciencesSol Plaatje University 473103 Kimberley 8301 South Africa; 2 School of Statistics and Actuarial ScienceUniversity of the Witwatersrand 37707 Johannesburg 2050 South Africa

**Keywords:** Bi-directional long-short memory, convolutional neural network, coronavirus deep learning, deoxyribonucleic acid, SARS-CoV-2

## Abstract

The SARS-CoV-2 virus which originated in Wuhan, China has since spread throughout the world and is affecting millions of people. When there is a novel virus outbreak, it is crucial to quickly determine if the epidemic is a result of the novel virus or a well-known virus. We propose a deep learning algorithm that uses a convolutional neural network (CNN) as well as a bi-directional long short-term memory (Bi-LSTM) neural network, for the classification of the severe acute respiratory syndrome coronavirus 2 (SARS CoV-2) amongst Coronaviruses. Besides, we classify whether a genome sequence contains candidate regulatory motifs or otherwise. Regulatory motifs bind to transcription factors. Transcription factors are responsible for the expression of genes. The experimental results show that at peak performance, the proposed convolutional neural network bi-directional long short-term memory (CNN-Bi-LSTM) model achieves a classification accuracy of 99.95%, area under curve receiver operating characteristic (AUC ROC) of 100.00%, a specificity of 99.97%, the sensitivity of 99.97%, Cohen’s Kappa equal to 0.9978, Mathews Correlation Coefficient (MCC) equal to 0.9978 for the classification of SARS CoV-2 amongst Coronaviruses. Also, the CNN-Bi-LSTM correctly detects whether a sequence has candidate regulatory motifs or binding-sites with a classification accuracy of 99.76%, AUC ROC of 100.00%, a specificity of 99.76%, a sensitivity of 99.76%, MCC equal to 0.9980, and Cohen’s Kappa of 0.9970 at peak performance. These results are encouraging enough to recognise deep learning algorithms as alternative avenues for detecting SARS CoV-2 as well as detecting regulatory motifs in the SARS CoV-2 genes.

**Nomenclature**
Abbreviation: Expansion
Acc: Accuracy
AUC: ROC Area Under Curve Receiver Operating Characteristic
Bi-LSTM: Bi-directional long short-term Memory
BPTT: Back-Propagation Through Time
CNN: Convolutional Neural Network
CNN-Bi-LSTM: Convolutional Neural Bi-directional
Long: Short-Term Memory
CoV: Coronavirus
COVID: Coronavirus Disease
DNN: Deoxyribonucleic Acid
FP: False Positive
FN: false-negative
LSTM: Long Short-term Memory
Kappa: Cohen’s Kappa
MCC: Mathew’s Correlation Coefficient
MLDSP-GUI: Machine Learning with Digital Signal Processing-Graphical User Interface
NCBI: National Centre for Biotechnology Information
NIR: No Information Rate
NLP: Natural Language Processing
RNA: Ribonucleic Acid
SARS: CoV-2 severe acute respiratory syndrome coronavirus 2
RNN: Recurrent Neural Network
RT-PCR: Reverse Transcription Polymerase Chain Reaction
TF: Transcription Factor
TN: True Negative
TP: True Positive
Sens: Sensitivity
Spec: Specificity
Prec: Precision

## I. Introduction

The SARS-CoV-2 virus which originated in Wuhan, China has since spread throughout all the provinces in China and the world and is affecting millions of people [1]. When there is a novel virus outbreak, it is crucial to quickly determine if the epidemic is a result of the novel virus or a well-known virus. This means that the proper classification of novel viruses such as SARS-CoV-2 and detecting regulatory or transcription motifs in these viruses can assist scientists in deciding on the methods and measures that are suitable to identify the viruses, control their transmission rates and limit potential consequences that may be caused by these viruses.

The identification of SARS-CoV-2 can give misleading results because the virus is hard to differentiate from other viruses in the *Coronaviridae* family, due to the genetic similarities among the viruses in this family [2]. This presents a challenge in that the detection of SARS CoV-2 viruses can yield false positives because of the presence of other viruses that are very similar to SARS CoV-2 [3]. Also, [3] states those patients who are suspected to have SARS-CoV-2 may present symptoms that are sometimes similar to a different respiratory viral infection. Therefore, it is of paramount importance to accurately characterise the SARS CoV-2 virus from similar viruses to enhance patient diagnostics and also manage the outbreak of SARS CoV-2 virus.

SARS-CoV-2 is spreading fast due to the lack of accuracy in the detection tools that are currently used in practice [2]. Besides, SARS-CoV-2 is a typical RNA virus that produces new mutations in a replication cycle of Coronavirus, with an average evolutionary rate of about 10^−4^ nucleotide substitutions per site each year [4]. This has brought into question the current techniques that are used to detect SARS-CoV-2. The reverse transcription-quantitative real-time polymerase chain reaction (RT-qPCR) is a molecular tool that is widely used in detecting SARS CoV-2 in patients. The RT-qPCR technique combines RT-PCR with qPCR to enable the measurement of RNA levels through the use of cDNA in a qPCR reaction [5]. According to [2], RT-qPCR has used ORF1ab and N genes to identify SARS CoV 2. Also, RT-qPCR has been questioned by [6] who report that the technique has achieved a negative rate of 17.8% when sputum samples were used in mild cases and 11.1% negative rate for severe cases. The techniques achieved negative rates of 26.7% and 27.0% in severe and mild cases respectively when applied on nasal swabs. In addition, the technique achieved negative rates of 40.0% and 38.7% in severe and mild cases respectively when applied on throat swabs. These variations may be a result of the variations that are present in the RNA sequences of the viral species [2]. Apart from giving false-negatives, the RT-qPCR technique can detect a small percentage of other similar Coronaviruses that may be present in a simple which may hinder the positive identification of SARS CoV-2 [2]. Furthermore, [7] indicates that about 35.2% of 173 samples did not test positive when the technique was used. Also, [8] report that real-time RT-PCR may initially produce false-negative results, and they suggested that patients with typical computed tomography (CT) findings, but negative real-time RT-PCR should repeat the real-time RT-PCR to avoid misdiagnosis.

As mentioned earlier, SARS CoV-2 is like other viruses in the *Coronaviridae* family, and its identification can be difficult. Therefore, we will explore how deep learning methods can be used to accurately identify SARS CoV-2 from other Coronaviruses. These methods can then be used to complement the existing molecular testing techniques to improve the detection rates of SARS CoV 2.

According to [9], motifs are approximate short nucleotide sequences that occur repetitively in similar groups of sequences. The regulatory motifs are used to control the expression of genes, i.e., they are responsible for turning a gene on or off. Also, transcription factors (TFs) are proteins that attach to DNA. The main function of TFs is to convert or transcribe DNA into Ribonucleic acid (RNA). TFs attach themselves to DNA sequences and become responsible for turning on or off genes through a process called “gene expression”. A particular TF binds to a specific site called a transcription factor binding site (TFBS), thus, regulates cell machinery [10].

It can be challenging in bioinformatics to identify regulatory motifs in DNA sequences [11]. This is because motifs are short sequences and their prediction usually results in several unacceptable false positives. In this paper, we will focus on regulatory motifs that are shared by the SARS CoV-2 genes in classifying whether a given sequence contains regulatory motifs for the SARS CoV-2 or not. Using deep learning, we focus on detecting nucleotides that are important in predicting whether a given sequence contains regulatory motifs for the SARS CoV-2 virus. The analysis of regulatory motifs is important for making improvements in medical treatment and gaining valuable knowledge about cell processes. For example, analysis of regulatory motifs may help better understand mutations that may affect the regulatory mechanism of gene expression.

We propose a hybrid deep learning algorithm that integrates a state-of-the-art CNN-Bi-LSTM to classify the SARS CoV 2 virus from other Coronaviruses as well as classify whether a given sequence contains regulatory motifs for the SARS CoV-2 or not. This paper makes the following specific contributions:

1) Develop an alignment-free method for classifying SARS-CoV-2 gene sequences amongst Coronaviruses’ genes,
2) Develop a deep learning algorithm that can efficiently classify whether a SARS CoV-2 genome sequence contains candidate regulatory motifs and
3) Compare the classification performances of our proposed CNN-Bi-LSTM versus the CNN and CNN-LSTM.

### A. Problem Statement

Detecting whether a given sequence contains regulatory motifs for the SARS-CoV-2 gene, as well as identification of SARS CoV-2 genes amongst Coronaviruses, can be viewed as binary classification problems in that we have a dataset $\mathcal {D}$ with ${N}$ examples of input data together with their corresponding target classes: $\mathcal {D} = \{\textbf {x}_{(i)}, y_{(i)}\}_{i=1}^{N}$, and **X** $\subset ~\mathbb {R^{d}}$ represents a feature space, which can be described as a matrix with dimensions, $4\times N$. The length of the DNA sequence is, thus, represented by ${N}$. We consider a value ${N} = 100$ base pairs (bp) in this paper. Additionally, $\textbf {Y} $ is a dichotomous variable in the standard space $\{ 0,1 \}$ [12]. As discussed earlier, there are four bases in DNA sequences namely: Adenine (A), Thymine (T), Guanine (G), and cytosine (C). These four base pairs form the sequence of base pairs {A, T, C, G} [12]. These base pairs can be characterised by one of the following one-hot vectors [1, 0, 0, 0], [0, 1, 0, 0], [0, 0, 1, 0] and [0, 0, 0, 1]. The SARS CoV-2 genes are like the other genes in the Coronavirus family [2], therefore, their classification can give rise to false results. Therefore, the major goal of this paper is to predict accurately SARS-CoV-2 gene sequences from amongst the Coronaviruses’ genes. Additionally, we classify whether a genome sequence contains candidate regulatory/promoter motifs for SARS CoV-2 genes.

## II. Related Work

Traditionally, the classification of genome sequences has used alignment-based techniques which include the Basic Local Alignment Search Tool (BLAST) [13] and the Burrows-Wheeler Aligner (BWA) [14]. Such techniques rely on annotating viral genes [15]. Alignment-based methods such as BLAST have been successful in finding sequence similarities [16]. However, in practice, these methods require heavy computational time when they are used to analyse thousands of complete genomes [17]. References [16], [18] mention that the alignments assume that the genes are homologous, i.e., they have the same continuous structure. However, in practice, this is not always the case.

Several alignment-free computational approaches [19], [20] have been used to predict deoxyribonucleic acid (DNA) protein binding. DeepFam which does not require the alignment of genes for predicting and modeling proteins was proposed by [21]. DeepFam uses a feedforward convolution neural network. It achieved better accuracy and faster run-time for predicting binding proteins when compared to methods that required the alignment of sequences as well as those that did not require the alignment of sequences [21]. Reference [18] proposed a Machine Learning with Digital Signal Processing-Graphical User Interface (MLDSP-GUI), which is an alignment-free tool for DNA sequence comparisons and analysis. The authors highlight that the tool was designed to address issues that are associated with the alignment of DNA sequences.

Our proposed model, CNN-Bi-LSTM is an alignment-free algorithm that consists of CNN layers followed by Bi-LSTM layers that capture the temporal effects in deoxyribonucleic acid (DNA) sequences [12]. DNA is made of nucleotide sequences whose function is to store information in all cells. Each nucleotide is made of sugar (Deoxyribose in DNA and Ribose in RNA), a base, and a phosphate. There are four bases in DNA sequences namely: Adenine (A), Thymine (T), Guanine (G), and cytosine (C). According to [12], these four base pairs form the sequence of base pairs {A, T, C, G}. We consider SARS CoV-2 gene sequences as patterns of letters made from the four nucleotides, A, T, G, and C, and then use one-hot vectors to represent these sequences in a similar way to text data. We adopt the procedure by [22] to translate DNA sequences into sequences of words. For example, [22] indicates that a dictionary of 64 words is formed when a word of size three nucleotides is used. This means that a one-hot vector of size 64 can represent every three-letter word. This method results in a sequence of words that can be represented by a two-dimensional matrix that encompasses information about the precise location of each base in the sequence. This numerical matrix is the input that is subsequently fed into a CNN. Additionally, one-hot vectors that are used in this paper to represent SARS CoV-2 gene sequences can conserve information about the position of each base in sequences [22].

The use of CNN is inspired by its successes in modelling DNA sequences. For example, [23] mention that CNNs have outperformed machine learning algorithms that include support vector machines (SVM) or random forests in predicting protein binding based on DNA sequences. Also, CNNs have been successfully used in DeepSea [24] to predict the chromatin effects sequence alterations with single nucleotide sensitivity. Besides, using patterns learned from experimental data, DeepBind has used CNN to discover specific DNA and RNA binding proteins [23]. The use of the CNN as part of an algorithm that can classify SARS CoV-2 gene sequences is also inspired by its successes in text classification [25]. Additionally, CNN has been used in topic categorisation [26], spam detection [27], and Twitter sentiment analysis [28].

Reference [22] states that one-dimensional sequences of successive letters can be used to represent text data. Therefore, one-hot vectors that are fed as input into CNN can be used to represent text data. Reference [26] recommend the use of one-hot vectors because the use of look-up tables that match each word in a word-vector is tantamount to using uni-grams information, whereas bi-grams and n-grams could be more discriminating in classifying samples. Thus, the use of one-hot vectors and concatenating word vectors of words that are close will include the n-gram information into text classification.

We use the CNN layers first to provide better input to the Bi-LSTM layers by generating filters that generalise sequence patterns [12]. The LSTM layers incorporate the long and short-term information that is present in DNA sequences [29]. The use of the Bi-LSTM layers is to ensure that we can utilise both past and future inputs i.e., DNA sequences at a given point in time. This means that the Bi-LSTM layer can make use of past and future DNA sequences by capturing the long-term relationships of a DNA sequence through the application of the forward LSTM as well as the backward LSTM. According to [12], the Bi-LSTM layer can characterise a probably very complex order in the DNA sequence in an efficient manner. Reference [12] developed DeepSite for predicting DNA-protein binding. DeepSite has Bi-LSTM network layer(s) followed by CNN layer(s). Reference [30] developed DanQ, similar to DeepSea, which is also uses CNN layers and Bi-LSTM layers for predicting the non-coding function at the start of a sequence. Our proposed model extends the work of [30] in classifying SARS CoV-2 gene sequences from amongst Coronaviruses as well as identifying sequences that contain regulatory motifs for the SARS CoV-2. Our model reverses the order of appearance of the Bi-LSTM and CNN layers in DeepSea.

## III. Materials and Methods

We propose a CNN-Bi-LSTM to classify SARS CoV-2 virus amongst coronaviruses and predict the short regulatory motifs (i.e., DNA binding motifs) that are bound to the proteins (transcription-factors). Our model is different from DeepSite [12] in that, we start with CNN layers that feed into Bi-LSTM layers. We employ the CNN-Bi-LSTM to extend the work by [2] to classify accurately SARS CoV-2 genes. Also, the CNN-Bi-LSTM extends the work of [20] to predict DNA binding motifs. Besides, combining CNN and Bi-LSTM layers is motivated by [31] who indicated that LSTMs performances can be improved by using CNN to provide better features to the LSTM.

### A. Datasets

The dataset for classifying SARS CoV-2 genes amongst Coronaviruses are summarised in Table 1. The dataset was obtained from the NCBI genes database on November 1, 2020.

**TABLE 1:** Data for Classifying SARS CoV-2 Genes Amongst Coronaviruses

Virus gene	Class Label	Number of Samples
SARS CoV-2	1	34
Non-SARS CoV-2	0	295

All repeating sequences were removed resulting in 329 unique sequences. All the virus genes belonged to the Coronavirus (CoV) family. We attached a label of 1 if a gene was that of SARS CoV-2 gene and 0 otherwise. The data was unbalanced with 10.3% positive SARS CoV-2 samples and 89.7% negative samples.

## IV. Algorithms

### A. Convolutional Neural Networks (CNN)

CNNs consist of a convolutional layer, a non-linearity layer, a max-pooling layer, and a fully connected layer [22]. CNNs have achieved outstanding performance in image classification, computer vision, and natural language processing (NLP) [32]. Also, they have been applied to text problems that include spam detection, sentiment classification and topic categorisation [33]. Text classification seeks to automatically classify text documents into one or more known categories. Text data is represented as a one-dimensional sequence of successive letters as opposed to image data which is represented as two-dimensional matrices. Therefore, if we are to use text data as an input in CNNs, we change the one-dimensional sequences of letters into a matrix or 2D tensor [26].

DNA sequences have patterns of successive letters that do not have space in contrast to text data which has space between words. These sequences are made up of “words” from the four nucleotides, A, T, G, and C [34]. The words formed by the sequences do not have any meaning. Reference [22] indicates that DNA sequences can be characterised using one-hot vectors into 2D matrices that are, then, fed into the next layer which in this work is a CNN layer. We will adopt the one-hot vectors proposed by [22], [26] to represent DNA sequences as 2D matrices.

A big argument for incorporating CNNs in our proposed model is that they are fast and efficient in terms of representation of text or sequences [35]. Thus, we use a deep learning algorithm that combines a CNN and Bi-LSTM for detecting sequences with regulatory or transcription motifs and also for the classification of SARS-CoV-2 genes amongst other Coronavirus genes.

### B. Long Short-Term Memory Network (LSTM)

Reference [36] introduced long short-term memory networks (LSTM) which are capable of learning long-term dependencies through recurrently connected memory blocks (subnets). Long short-term memory networks (LSTMs) are an example of recurrent neural networks (RNN) [36]. RNNs described in detail in [37] are deep neural networks that can process sequential data where outputs are dependent on the previous computations. However, RNNs are easily affected by the vanishing gradients problem [38]. Thus, RNNs become biased as they only deal with short-term data points. For time or sequence-dependent data, an RNN takes the output of a layer at time *t* and feeds it as part of the input of a layer at time ${t} + 1$. LSTM operates above the RNN and they add some memory components that assist in propagating the knowledge learned at a time ${t}$ to the longer-term time-steps, (e.g, ${t} + 1$, ${t} + 2,\ldots $). The most important function of an LSTM is to overlook insignificant parts of the preceding state, carefully update a current state, and then output only important parts of the current state that are required in future states. This solves the vanishing gradient problem in RNNs by updating a state then propagating forward important parts of that state that are pertinent to future states. Thus, LSTMs become far more efficient than RNNs as there is not an extended back-propagation chain often seen in RNNs [36].

**FIGURE 1.**
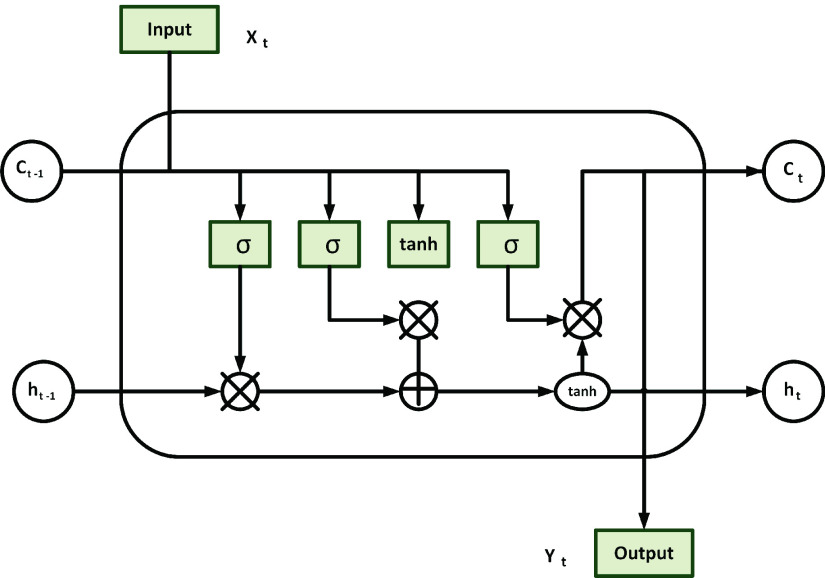
Schematic representation of a LSTM cell.

LSTMs use the input gate, forget gate, and output gate to release information between the hidden state and the cell state. The structure of an LSTM cell is shown in Fig. 1, where $X_{t}$: input vector, $h_{t}$: output of the current network, $h_{t-1}$: output from previous LSTM unit, $C_{t-1}$: a memory of the previous unit, $C_{t}$: a memory of the current unit, $\bigotimes $: element-wise multiplication, $\bigoplus $: element-wise summation and tanh: the hyperbolic tangent.

Fig. 1 shows that an LSTM unit is made up of a cell, with a state $C_{t}$ over time. The LSTM unit uses the following gates: input $I_{t}$, output, $O_{t}$ and forget, $f_{t}$ gates for modifying and adding memory in the cell. The flow of information into the cell as well as out of a cell is controlled by these three gates. Also, a cell emits $h_{t}$, an output signal after updating a gate. To update $h_{t}$, the sigmoid layer of an LSTM cell unit is initialised at the forget gate, $f_{t}$. Then, the LSTM cell unit determines the importance of $C_{t-1}$. Consequently, the sigmoid layer (“input gate layer”) chooses the values to update. After that, a vector of new candidate values, $\tilde {C_{t}}$ is created using the tanh layer. $\tilde {C_{t}}$ may be appended to the state $C_{t-1}$, simultaneously, removing or forgetting some values. Moreover, multiplying $C_{t-1}$ by $f_{t}$ (without the removed or “forgotten values”) and then adding $I_{t}~\cdot ~\tilde {C_{t}}$ updates $C_{t}$. Thus, $I_{t}~\cdot ~\tilde {C_{t}}$ is made up of the new candidate values multiplied by the input values of the current state. Lastly, the output of the LSTM cell is computed by employing the third sigmoid level along with another tanh filter [39]. The following equations [39], [40] summarise the process of obtaining the output of the hidden state, $h_{t}$;

\begin{align*} f_{t}=&\sigma (\boldsymbol {W}_{f} [h_{t-1}, x_{t}] + b_{f}), \tag{1}\\ I_{t}=&\sigma (\boldsymbol {W}_{i} [h_{t-1}, x_{t}] + b_{I}), \tag{2}\\ \tilde {C_{t}}=&\tanh (\boldsymbol {W}_{C} [h_{t-1}, x_{t}] + b_{C}), \tag{3}\\ C_{t}=&f_{t} \cdot C_{t-1} +I_{t} \cdot \tilde {C_{t}}, \tag{4}\\ o_{t}=&\sigma (\boldsymbol {W}_{i} [h_{t-1}, x_{t}] + b_{o}), \tag{5}\\ h_{t}=&o_{t} \cdot \tanh (C_{t}).\tag{6}\end{align*}

$C_{0} = 0 $ and $h_{0} = 0$, indicate initial values, and $t$ represents the time steps. The activation function is represented by, $\sigma $. It takes values between 0 to 1, thereby, ensuring that the data is removed completely, partially removed, or preserved. $\tilde {C_{t}}$ is a “candidate” hidden state. Its values are updated using the current input value and the previous hidden state’s value. $I_{t}$ is an input gate that controls the amount of information from the newly computed current state that is allowed to pass through, $h_{t-1}$ connects the previously hidden layer and the current hidden layer recurrently, $\boldsymbol {W}$ represents the weight matrix that connects the inputs to the current hidden layer, the internal memory of a cell unit is represented by $C_{t}$, and the output of a hidden state is given by $h_{t}$.

The LSTM neural network uses the activation functions, tanh and sigmoid. Neural networks use these activation functions to learn complex data patterns. They work by converting the output signal from a previous cell into a form that serves as the input to the next cell. Also, they add non-linearity in data to make it similar to real-world data or problems [40], [41]. Ideally, tanh is used in situations where signals from historical data points are required because it can sustain information for a longer period before going to zero [40]. Also, Fig. 1 shows that we need another activation function called the sigmoid function to either forget or recall some of the information.

We use LSTM networks as they are capable of learning long-term dependencies through recurrently connected subnets known as memory blocks [42]. LSTM networks can learn complex structures within the sequential ordering of sequences. Besides, they utilise internal memory to remember information across long input sequences. Long short-term memory (LSTM) networks are designed to solve the vanishing gradient problem associated with RNNs.

### C. Bi-Directional Long-Term Memory Recurrent Neural Network (Bi-LSTM)

The LSTM addresses the problem of long-time lags found in RNNs. There are situations where predictions have to be made by looking at both the prior and subsequent inputs. The bidirectional LSTM (Bi-LSTM) proposed by [36] addresses the problem of making predictions based on previous and subsequent inputs.

**FIGURE 2.**
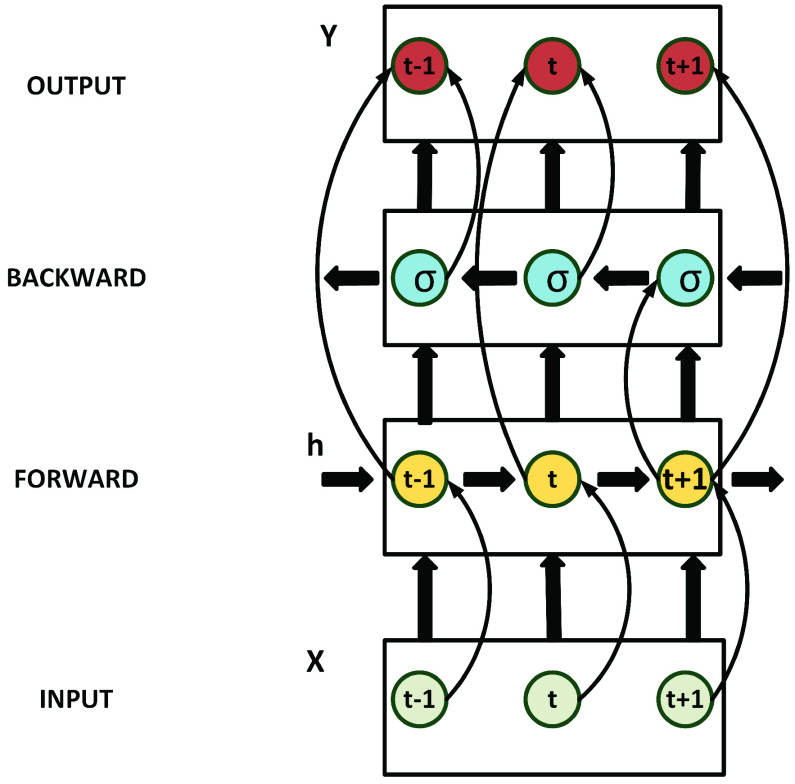
Schematic representation of a Bi-LSTM.

Fig. 2 shows that the Bi-LSTM has a forward layer that first calculates the network from time ${T} = 1$ to time ${T} = {t}$.

The hidden layers’ output at each time-step from ${T} = 1$ to time-step ${T} = {t}$ is saved. Then a reverse calculation of the network using a backward layer occurs and the outcome of the hidden layer at each time from time-step ${t}$ to time-step 1 is calculated and saved [43]. Reference [44] mentions that the outputs of the forward and backward layer are then combined at each time step using one of the following means: (i) Concat: Where the outputs are concatenated together. (ii) Mul: Where the outputs are multiplied together, (iii) Sum: Where the outputs are added and (iv) Ave: Where the average of the two outputs is taken.

We implement concat in our proposed model to merge the outputs from the forward and backyard layers as it is the default method often used in bidirectional LSTMs [44]–[50]. Besides, concat doubles the output vector size that serves as input to the next layer [44], and this will result in better performance or a lower log loss. We train our proposed model using the Backpropagation Through Time (BPTT) algorithm [51] to resolve the problem of the vanishing/exploding gradient.

## V. Proposed Architecture

**FIGURE 3.**
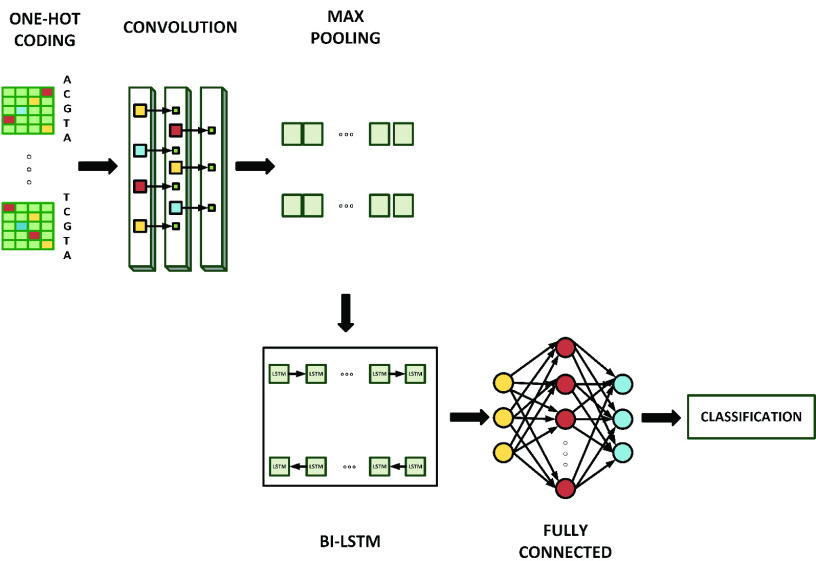
Schematic representation of the CNN-Bi-LSTM.

Fig. 3 shows the architecture of the CNN-Bi-LSTM that uses CNN layers as well as max-pooling layers for extracting features from input data, combined with a bi-directional LSTM network for interpreting the features across time steps and also perform sequence prediction. The proposed CNN-Bi-LSTM will consist of three CNN layers, then a Bi-LSTM layer and a dense layer as the output. Also, the architecture includes dropout layers that are deployed to address the problem of over-fitting that is common in deep neural networks [12]. Our proposed architecture follows the suggestions made by [22], [26] in that, we replace the coding/encoding layer and embedding layers by directly applying the CNN to high-dimensional one-hot vectors; i.e., embeddings of text regions are directly learned without going through the word embedding learning process. Also, we utilise one Bi-LSTM layer.

### A. Experiments

We carried out experiments to determine the classification performance of the CNN-Bi-LSTM algorithm on the SARS CoV-2 dataset described in Section III. For deep learning methods, pre-processing of data is very important. We created class labels to indicate whether a genome sequence was that of SARS-CoV-2 (positive samples) or not (negative samples). From the NCBI genes database, we obtained 34 positive samples all of which were marked as SARS-CoV-2 gene sequences (Table 1). Also, we obtained 295 negative samples, none of which was marked as SARS CoV-2 gene sequences.

We used **Keras** [52] to define the CNN-Bi-LSTM model by first creating the CNN layers, then the Bi-LSTM layers and output layers. The CNN-Bi-LSTM model was trained to classify SARS-CoV-2 virus sequences amongst Coronaviruses’; as well as classify whether a virus gene sequence contains SARS CoV-2 regulatory motifs or not. The deep learning models were trained independently using batch sizes of 64 as recommended by [12], [53]. We used Kera’s default weights and biases. The models are trained for 100 epochs using the recommended default learning rate, $\text {lr} = 0.001$ [12], [54]. We used dropout ratios equal to 0.1, 0.3, and 0.5. Following [12], we changed the number of cells in the Bi-LSTM layer from 32 to 256 and set the default number of cells to 32. The number of filters in the CNN layers is changed from 32 to 256 and we used a default value of 32 filters. Additionally, we utilised the binary log-loss (binary cross-entropy) and the efficient **Adam** [54] optimisation algorithm. The output layer was a fully connected layer with sigmoid as the activation function to perform binary classification [12]. Finally, we evaluated the skill of deep learning models. Deep learning algorithms are stochastic and have some additional sources of variation. The additional randomness allows model flexibility during the learning phase. However, this flexibility can make the model be unstable i.e., producing different results when the model is trained on the same data. To mitigate this problem, we carried out 100 iterations of each experiment and then took the average of the evaluation metrics for 100 iterations. Each model was trained for 100 epochs.

## VI. Results

The most commonly used model evaluation metric for binary classification is accuracy which can be misleading when used as the only performance metric in the case where the data is unbalanced. The data for classifying SARS CoV-2 genes was unbalanced with 10.3% positive and 89.7% negative samples. The dataset for classifying virus genes with regulatory motifs for the SARS CoV-2 genes was unbalanced with 3.69% positive samples (with regulatory motifs) and 96.31% negative samples. This means that classification may not work well as the classifiers may be biased towards the majority class. Therefore, the deep learning models are evaluated and compared by making use of a confusion matrix and then deriving the following metrics:

(i) Sensitivity (Sens)
  \begin{equation*} =\rm {\frac {TP}{TP + FN}}\end{equation*}
(ii) Specificity (Spec)
  \begin{equation*} = \rm {\frac {TN}{TN + FP}}\end{equation*}
(iii) Precision (Prec)
  \begin{equation*} = \rm {\frac {TP}{TP + FP}}\end{equation*}
(iv) Accuracy (Acc)
  \begin{equation*} = \rm {\frac {TP +TN}{TP + FP + FN + FP}}\end{equation*}
(v) Mathew’s Correlation Coefficient (MCC)
  \begin{equation*} = \rm {\frac {TP \cdot \hbox {TN-FN} \cdot FP}{\sqrt {(TP + FN)(TP + FP)(TN + FN)(TN + FP)}}}\end{equation*}
  where TP represents the true positives, TN represents the true negatives, FP and FN represent the false positives and false-negatives, respectively. Reference [55] states that MCC in the interval [−1, 1], with 1 indicating that there is perfect classification, -1 indicating a perfect misclassification.
(vi) Cohen’s Kappa ($\kappa $): is a robust statistic that can be used to assess the performance of classifiers. Also, Kappa considers a model’s accuracy obtained by chance. $\kappa $ can be calculated using; $\kappa = \frac {O - E}{1 - E} $ [56], where ${O}$ is the accuracy that is observed and ${E}$ is the expected accuracy. In this paper, we will use Cohen’s Kappa to assess the performances of our algorithms when performing classification tasks. $\kappa $ is similar to correlation coefficients and takes values from -1 to +1 inclusive; where a value of 0 means that the predicted class and observed class do not agree, while a value of 1 indicates that the observed class and the predicted class agree perfectly [56]. Also, [57] states that $\kappa $ values less than 0.20 indicate poor agreement, values between 0.20 - 0.40 indicate fair agreement, values between 0.40 - 0.60 indicate moderate agreement whilst substantial agreement starts at a value of 0.61. Excellent examples and explanations on the use of Cohen’s Kappa for classification can be found in [56]. Besides, [58] provides a caret R package for computing Cohen’s Kappa. The most commonly used model evaluation metric for binary classification is accuracy which can be misleading when used as the only performance metric in the case where the data is unbalanced. The data for classifying SARS CoV-2 genes was unbalanced with 10.3% positive and 89.7% negative samples. The dataset for classifying virus genes with regulatory motifs for the SARS CoV-2 genes was unbalanced with 3.69% positive samples (with regulatory motifs) and 96.31% negative samples. This means that classification may not work well as the classifiers may be biased towards the majority class. Therefore, we will use Cohen’s Kappa to evaluate how the actual classes and the classes predicted by the CNN-Bi-LSTM, CNN-LSTM, and CNN models agree.
(vii) No information Rate (NIR) and ${P}$-Value [Acc > NIR]. A good model is one where the accuracy is significantly greater than the no information rate. This means that a model with an accuracy that is less than the NIR is poor at classifying imbalanced data as it is just predicting the majority class most of the time. Such a model is said to be unreliable [59]. Besides, the model is also said to be poor if the rate of the majority class equals the classification accuracy. Therefore, a hypothesis test is carried out to assess if the overall accuracy rate is greater than the rate of the majority class (NIR), i.e., ${P}$-Value [Acc > NIR]. A significant ${P}$-value [Acc > NIR] indicates that our model is better than just classifying all into the majority class.

In addition to the metrics above, the predictive performance of each deep learning model is assessed using the AUC ROC.

### A. Parameter Analysis

#### 1) Performance Comparison Using Different Learning Rates

To obtain optimal performance for classifying SARS CoV-2, the hyper-parameters of our deep learning algorithms need to be tuned. The learning rate (lr) is an important hyper-parameter that has to be tuned for the deep learning algorithms to obtain optimal results. Reference [12] state with a lower lr, the training phase of the deep learning algorithm becomes more reliable. However, a lower lr may come at the expense of taking much time during the optimisation phase as the updated values of the loss function may be small [12]. A higher lr may cause the training stage not to converge and it even diverges [12]. Also, [12] mentions that with a higher learning rate, the optimisation phase may skip the optimal value, and the optimisation phase of the loss function may become even worse. Thus, there is a risk of skipping the optimal value when using a larger learning rate and this may adversely affect the accuracy of the algorithm [60]. This is because a larger learning rate requires more training time as it is continually skipping the optimal value and “unlearning” what has already been learned, resulting in unproductive oscillations of the accuracy. These oscillations will cause poor generalisation of the accuracy because the training weights never settle down to give an optimal value (minimum). As recommended by [12], we used the (default) learning rate, $\text {lr} = 0.001$ for the Adam algorithm for stochastic optimisation to update the parameters. Moreover, [54] states that a default $\text {lr} = 0.001$ for the Adam optimiser is a good learning rate for stochastic optimisers.

#### 2) Performance Comparison Using Different Dropout Ratios

Deep neural networks with many parameters may suffer from the problem of over-fitting. To address this problem, we use the dropout technique described in detail in [61]. The dropout technique temporarily removes a hidden and or a visible unit together with all its incoming and outgoing connections. The units that are selected to be dropped out are selected at random. In this paper, we investigate the effect of the dropout technique in preventing over-fitting and improving accuracy. We applied dropouts after the convolutional and max-pooling layers as well as in the LSTM cell implementation. Tables 2 and 3 show that the performance of our proposed model (CNN-Bi-LSTM) is similar and stable for dropout ratios 0.1 and 0.3. However, the performance drops slightly when the dropout ratio is set to 0.5. Probably, this shows that a higher dropout of 0.5 maybe resulting in a higher variance to some of the layers, and this has the effect of degrading training and, reducing performance. Thus, at a 0.5 dropout ratio, the capacity of our model is marginally diminished causing the performance of the model to marginally deteriorate. Therefore, for the sake of comparison, we specify a dropout ratio of 0.1 for implementation in the CNN, CNN-LSTM, and CNN-Bi-LSTM models.

**TABLE 2:** A Comparison of CNN-BiLSTM’s Performance With Changing Dropout Ratios

Dropout ratio	Precision(%)	Specificity(%)	Sensitivity(%)
0.1	99.81	99.97	99.97
0.3	99.81	99.97	99.97
0.5	99.26	98.33	98.33

**TABLE 3:** A Comparison of CNN-BiLSTM’s Performance With Changing Dropout Ratios

Dropout ratio	AUC ROC(%)	Acc(%)	MCC	Kappa
0.1	99.81	99.95	0.9782	0.9975
0.3	99.00	99.94	0.9596	0.9775
0.5	99.91	99.9	0.9782	0.9667

#### 3) Performance Comparison Using Different Numbers of Convolutional Filters in CNN

We gradually varied the number of filters or kernels in CNN from 32, 64, 128 to 256. By varying the number of kernels or filters in CNN, we were able to evaluate Sens, Spec, Acc, Prec, MCC, AUC ROC, and Cohen’ Kappa values on the training dataset. Table 4 shows how the evaluation metrics vary under different numbers of convolutional filters. We see that the values of Sens, Spec, Acc, Prec for the CNN-Bi-LSTM model are slightly higher than those of the CNN-LSTM and CNN models. Also, we observe that the AUC ROC values for the CNN-Bi-LSTM model are superior to those of the other models as the number of convolutional filters increases. This indicates that our proposed model outperforms the CNN-LSTM and the CNN models. Specifically, the AUC ROC for the CNN-Bi-LSTM model improves considerably as the number of filters increases from 32 to 128. Table 4 shows that when the number of filters is equal to 32, the CNN-Bi-LSTM model performs marginally better than the CNN-LSTM and CNN models in all metrics. For example, when the number of convolutional filters is 32, the values of Sens, Spec, Prec, Acc, AUC ROC, MCC, and Kappa for our proposed model are 99.97%, 99.97%, 99.91%, 99.95%, 99.81%, 0.9978, and 0.9978, respectively. These results show that the performance of the CNN-Bi-LSTM is comparable to that of the CNN-LSTM model and performs marginally better by gaps of 1.01%, 1.01%, 0.65%, 0.3%, 6.27%, 0.0159%, and 0.0164% respectively. Similarly, our proposed model’s performance is comparable to that of the CNN model and performs marginally better by gaps of 1.43%, 1.43%, 0.09%, 0.30%, 8.64%, 0.00%, and 0.024% respectively. Therefore, for the sake of comparison, we use the default 32 cells in the convolutional layers of all three models.

**TABLE 4:** Performance Comparison Using Different Numbers of Filters in CNN

	Cell numbers	CNN-Bi-LSTM	CNN-LSTM	CNN
Sens (%)	32	99.97	98.96	98.54
	64	99.16	96.52	99.38
	128	99.83	97.71	97.91
	256	99.97	97.71	99.33
Spec (%)	32	99.97	98.96	98.54
	64	99.16	96.52	99.38
	128	99.97	97.71	97.91
	256	99.97	97.71	99.33
Prec(%)	32	99.91	99.26	99.82
	64	99.92	99.26	99.92
	128	99.83	99.26	100.0
	256	99.82	99.26	99.95
Acc (%)	32	99.95	99.65	99.65
	64	99.85	99.44	99.84
	128	99.95	98.19	99.39
	256	99.95	99.74	99.89
AUC ROC (%)	32	99.81	93.54	91.17
	64	100.0	91.80	96.46
	128	100.0	93.91	94.67
	256	99.52	92.21	94.75
MCC	32	0.9978	0.9819	0.9778
	64	0.9782	0.9819	0.9964
	128	0.9782	0.9819	1.000
	256	0.9978	0.9819	0.9921
Cohen’s Kappa	32	0.9978	0.9814	0.9734
	64	0.9882	0.9380	0.9915
	128	0.988	0.9582	0.9614
	256	0.9978	0.9582	0.9912

#### 4) Performance Comparison Using Different Numbers of Cells in LSTM

We carried out experiments with different numbers of cells in the LSTM part of the model to choose the optimal number of cells that improves the performances of the deep learning algorithms. By varying the numbers of cells from 32, 64, 128 to 256, we were able to evaluate Sens, Spec, Prec, Acc, MCC, AUC ROC, NIR and Cohen’ Kappa values on the training dataset. Table 5 shows the performances of the CNN-Bi-LSTM and CNN-LSTM with a different number of cells in the LSTM. The results show that Sens, Spec, Prec and Acc for our proposed model are generally higher than those of the CNN-LSTM model. The AUC ROC of our proposed model significantly increases when the number of cells changes from 32 to 128 and then stabilises when the number of cells is 256. Furthermore, Table 5 shows that the best performing number of cells in the LSTM is 32. The values of Sens, Spec, Prec, Acc, AUC ROC, MCC, and Kappa for the CNN-Bi-LSTM model when the number of cells is 32 are: 99.97%, 99.97%, 99.81%, 99.95%, 99.81%, 0.9978, and 0.9978, respectively. These values show that our proposed model outperforms the CNN-LSTM model by gaps of 1.01%, 1.01%, 0.55%, 0.3%, 6.27%, 0.0159, and 0.0164 respectively. Therefore, for the sake of comparison, we use the default 32 cells in the LSTM layers.

**TABLE 5:** Performance Comparison Using Different Numbers of Cells in LSTM

	Cell numbers	CNN-Bi-LSTM	CNN-LSTM
Sens (%)	32	99.97	98.96
	64	99.94	99.56
	128	97.96	99.28
	256	99.92	99.20
Spec (%)	32	99.97	98.96
	64	99.94	99.56
	128	97.96	99.26
	256	99.92	99.2
Prec(%)	32	99.81	99.26
	64	99.63	99.74
	128	99.58	99.61
	256	99.44	99.07
Acc (%)	32	99.95	99.65
	64	99.90	99.90
	128	99.49	99.80
	256	99.85	99.70
AUC ROC (%)	32	99.81	93.54
	64	99.91	93.59
	128	1.000	93.62
	256	99.99	91.84
MCC	32	0.9978	0.9819
	64	0.9956	0.9926
	128	0.9691	0.9880
	256	0.9932	0.9811
Cohen’ Kappa	32	0.9978	0.9814
	64	0.9955	0.9921
	128	0.9639	0.9869
	256	0.9925	0.9791

#### 5) Model Training Time

We also consider the cost in terms of the time each model takes to train for 100 epochs, i.e., the time it takes to complete 100 training epochs as shown in Table 6.

**TABLE 6:** Model Total Training Time for 100 Epochs

Models	Trainable Parameters	Training epochs	Training time(s)
CNN-Bi-LSTM	27 892	100	166.33
CNN-LSTM	17 268	100	98.1442
CNN	31 394	100	71.4451

Table 6 shows that adding a Bi-LSTM layer after the CNN layers results in the proposed model taking much more time to train for 100 epochs than the CNN-LSTM and CNN models. Moreover, the results show that the additional time taken by CNN-Bi-LSTM offers marginally better performance than the CNN-LSTM and CNN models because the Bi-LSTM layer has additional training capabilities [62].

### B. Performance Comparison

#### 1) Performance Comparison of CNN-Bi-LSTM, CNN-LSTM and CNN Models

Using the results from Table 4, we evaluated the peak performances of the three models. Table 7 displays the *peak* performance comparisons of the three models when they are used to classify SARS CoV-2 virus amongst Coronaviruses. Our proposed model is comparable and achieves similar performances to those of the other models in almost all the evaluation metrics. The results show that the CNN-Bi-LSTM achieves 99.97%, 99.97%, 99.92%, 99.95%, 100.0%, and 0.9978 for Sens, Spec, Prec, Acc, AUC ROC, and Cohen’s Kappa, respectively. These values show that at the peak, our proposed model’s performance is marginally higher than that of the CNN-LSTM model by gaps of 1.01%, 1.01%, 0.66%, 0.21%, 6.09%, and 0.0063 respectively for Sens, Spec, Prec, Acc, AUC ROC, and Cohen’s Kappa. Similarly, our proposed model’s performance is marginally higher than that of the CNN model by gaps of 0.59%, 0.59%, 0.06%, 0.54%, and 0.0063 respectively for Sens, Spec, Acc, AUC ROC, and Cohen’s Kappa. These results show that the CNN-Bi-LSTM that combines the CNN and Bi-LSTM layers marginally improves performance compared to the other models. Furthermore, these results demonstrate the added advantage of using the Bi-LSTM layer which incorporates both previous input values and future input values.

**TABLE 7:** Peak Performance Comparisons in the Classification of SARS CoV-2 Amongst Coronaviruses

	CNN-Bi-LSTM	CNN-LSTM	CNN
Sens (%)	99.97	99.86	99.38
Spec (%)	99.97	99.86	99.38
Prec(%)	99.92	99.26	100
Acc (%)	99.95	99.74	99.89
AUC ROC (%)	100.00	93.91	99.46
MCC	0.9978	0.9819	1.000
Cohen’s Kappa	0.9978	0.9814	0.9915

#### 2) Approximate Statistical Tests for Comparing the CNN-Bi-LSTM, CNN-LSTM, and CNN Models

Table 7 shows that the peak performances of our proposed model are comparable and in some cases marginally higher than those of the CNN-LSTM and CNN models. However, there is a need to perform hypothesis tests that can spot any differences better than the human eye to examine if the differences in the performance of the models are statistically significant. Thus, we applied the post-hoc $5 \times 2$-fold cv paired ${t}$-test as opposed to the k-fold cross-validated paired ${t}$-test [63] to test for the differences in performance relative to the AUC ROC. The k-fold cross-validation is widely used to evaluate the performance of different models by computing and directly comparing different performance metrics [64]. However, in the k-fold cross-validated paired ${t}$-test, the training data sets may overlap. For example, in 10-fold cross-validation, each pair of the training data sets shares 80% of the data examples. This presents a problem as the overlap may prevent the paired ${t}$-test from obtaining good estimates of the amount of the variation that would have been accounted for had the training data sets been entirely independent of the other previous training data sets [63]. Also, [63], mentions that the 10-fold cross-validation technique shows higher probabilities of type 1 errors. To solve the problem where the training data sets may overlap, [63] recommended using a $5 \times 2$-fold cv paired ${t}$-test which is based on repeating two-fold cross-validations five times. The two-fold cross-validation is used because it yields larger test data sets as well as training data sets that are disjoint. The $5 \times 2$-fold cv paired ${t}$-test is a more powerful test than the k-fold cross-validated paired t-test as it directly measures variation that is due to the choice of the training data set. Thus, we use the $5 \times 2$-fold cv paired ${t}$-test to perform a post-hoc analysis to determine the statistical significance of the differences in the means of the performance metric scores. Following [12], [65], we chose the AUC ROC as a specific measure to choose the model that would be more accurate on new test data. The test statistic $\tilde {t}$, for the $5 \times 2$-fold cv paired ${t}$-test is calculated using the following equation [63]

\begin{equation*} \tilde {t} = \frac {p_{1}^{(1) }}{\sqrt {\frac {1}{5}\sum _{i = 1}^{5}s_{i}^{2}}}\tag{7}\end{equation*}

where $p_{1}^{(1) }$ is the difference in the AUC ROC scores of the CNN-Bi-LSTM vs CNN or CNN-LSTM models for the first fold of the first iteration, $s_{i}^{2}$ is the variance of the AUC ROC score differences of the ${i}$th iteration. The variance is computed using; $\left ({p_{i}^{(1) } - \bar {p_{i}}}\right)^{2} + \left ({p_{i}^{(2) } - \bar {p_{i}}}\right)^{2}$. In addition, $p_{i}^{(j)}$ is the difference in the AUC ROC scores of the CNN-Bi-LSTM vs CNN or CNN-LSTM models for the ${i}$th iteration and fold ${j}$ and $\bar {p_{i}} = \left ({p_{i}^{(1) } + p_{i}^{(2) }}\right)/2$.

Under $H_{0}$, $\tilde {t}$ approximately follows a ${t}$ distribution with 5 degrees of freedom. We let $H_{0}$, be such that there is no statistically significant difference between the AUC ROC of the CNN-Bi-LSTM vs CNN or CNN-LSTM models and $H_{1}$, the alternative hypothesis, such that there is a statistically significant difference between the AUC ROC of the CNN-Bi-LSTM vs CNN or CNN-LSTM models. Accepting the null hypothesis, $H_{0}$, for a given level of significance would mean that the differences in the estimated performance metrics are due to chance. However, if $H_{0}$ is rejected, we conclude that any differences in the performance metrics are due to the differences in the models.

Table 8 shows the post-hoc statistical analysis, using the $5 \times 2$-fold cv paired ${t}$-test relative to the AUC ROC performance metric for the CNN-Bi-LSTM versus the CNN models. The $5 \times 2$ cv Paired ${t}$-test from Table 8 produced a ${t}\text {-value} = 3.877$. This ${t}$-value is assumed to follow a ${t}$-distribution with 5 degrees of freedom. Thus, the critical value, $t_{5, 0.975} = 2.571$. Since ${t}\,\,\text {value} = 3.877 > t_{5, 0.975} = 2.571$, we conclude that the differences in the AUC ROC scores are due to the differences in the performance of the CNN-Bi-LSTM and CNN models. Thus, the CNN-Bi-LSTM outperforms the CNN model relative to the AUC ROC.

**TABLE 8:** $5 \times2$ cv Paired t-Test for the CNN-Bi-LSTM and the CNN Models Relative to the AUC ROC

Folds	CNN-Bi-LSTM Scores	CNN Scores	Scores differences
Fold 1	98.89	93.06	5.83
Fold 2	100	98.89	1.11
Fold 1	100	99.42	0.58
Fold 2	100	99.83	0.17
Fold 1	100	100	0
Fold 2	100	100	0
Fold 1	100	100	0
Fold 2	100	100	0
Fold 1	100	100	0
Fold 2	100	100	0
Mean	99.89	99.01	0.869
stdev	0.333	2.038	1.745

Table 9 shows the post-hoc statistical analysis, using the $5 \times 2$-fold cv paired ${t}$-test relative to the AUC ROC performance metric for the CNN-Bi-LSTM versus the CNN-LSTM models. The $5 \times 2$ cv Paired ${t}$-test from Table 9 produced a ${t} -\text {value} = 3.654$. The critical value, $t_{5, 0.975} = 2.571$. Since ${t}\,\,\text {value} = 3.654 > t_{5, 0.975} = 2.571$, we conclude that the differences in the AUC ROC scores are statistically significant and are due to the differences in performance of the CNN-Bi-LSTM and CNN-LSTM models. The results show that relative to the AUC ROC, the CNN-Bi-LSTM performs better than the CNN-LSTM.

**TABLE 9:** $5 \times2$ cv Paired t-Test for the CNN-Bi-LSTM and the CNN-LSTM Models Relative to the AUC ROC

Folds	CNN-Bi-LSTM Scores	CNN-LSTM Scores	Scores differences
Fold 1	75.83	74.44	1.39
Fold 2	100	98.89	1.11
Fold 1	99.42	98.26	1.16
Fold 2	100	100	0
Fold 1	100	100	0
Fold 2	100	100	0
Fold 1	100	100	0
Fold 2	100	100	0
Fold 1	100	100	0
Fold 2	100	100	0
Mean	97.52	97.16	0.367
stdev	7.233	7.594	0.564

#### 3) Performance Comparison of the CNN-Bi-LSTM with Different Datasets

To evaluate the performance of the proposed CNN-Bi-LSTM model on new data, we conducted experiments using different datasets with 25%, 50%, 75%, and 100% of the dataset with regulatory motifs for the SARS CoV-2 gene sequences obtained from the NCBI database. Table 10 shows the genes with regulatory motifs for the SARS CoV-2 discovered by [9].

**TABLE 10:** Genes With Regulatory Motifs for the SARS CoV-2

Name /Gene ID	Description
orf8/43740577	orf8 protein
orf10/43740576	orf10 protein
N/43740575	Nucleocapsid phosphoprotein
orf7b/43740574	orf7b protein
orf7a/43740573	orf7a protein
orf6/43740572	orf6 protein
M/43740571	Membrane glycoprotein
E/43740570	Envelope protein
orf3a/43740569	orf3a protein
S/43740568	Surface glycoprotein

Reference [9] analysed whether the following eleven genes had regulatory motifs for SARS-CoV-2 virus: orf1ab/43740578, orf8/43740577, orf10/43740576, N/ 43740575, orf7b/43740574, orf7a/43740573, orf6/43740572, M/43740571, E/43740570, orf3a/43740569 and S/43740568, using MEME [66]. The searches were done to identify common candidate regulatory motifs that serve as positions where transcription factors (TFs) can bind to. In turn, TFs control the expression of the SARS CoV-2 genes [9]. The authors found out that ten of these genes except the orf1ab/43740578 gene had DNA sequences that were responsible for turning on/off the SARS CoV-2 genes. All the genes that contained the regulatory motifs for the SARS CoV-2 were attached to label 1. Also, the gene orf1ab/43740578 is present in SARS CoV-2 genes but it was attached to the label 0 as it does not have regulatory motifs for the SARS CoV-2 genes [9]. Also, all other genes from the *Coronaviridae* family that do not contain regulatory motifs for the SARS CoV-2 genes were attached to the label 0.

The data for classifying whether a virus gene contains regulatory motifs for the SARS CoV-2 genes was organised and summarised as shown in Table 11.

**TABLE 11:** Data for Classifying Whether a Virus Gene Contains Regulatory Motifs for the SARS CoV-2 Genes

Virus gene	Class Label	Number of Samples
With regulatory motifs	1	76
Without regulatory motifs	0	1982

Table 11 shows that the dataset is unbalanced with 3.69% positive samples (with regulatory motifs) and 96.31% negative samples. We used 80% of the dataset for training and 20% for testing. Based on the experimental results in Section IV, we extracted the parameters shown in Table 12. With these parameter settings, we performed experiments using the different fractions of the dataset to evaluate the performance of the CNN-Bi-LSTM.

**TABLE 12:** Optimum Parameter Settings for the CNN-Bi-LSTM, CNN-LSTM and CNN Models

Parameter	CNN-Bi-LSTM	CNN-LSTM	CNN
Learning rate	0.001	0.001	0.001
Dropout ratio	0.1	0.1	0.1
Number of Kernels	32	32	32
Number of Cells	32	32	–
Epochs	100	100	100
Batch size	64	64	64
Number of Iterations	100	100	100

Table 13 shows that the performance of the CNN-Bi-LSTM remains excellent when applied to a new dataset. The new dataset is used to classify whether a virus gene contains regulatory motifs for the SARS CoV-2 genes or not. Additionally, we find out that as the cardinality of the data increases, the AUC ROC increases. This shows that our model’s performance improves with more data. At 100% the size of our dataset, there is more training data that the CNN-Bi-LSTM effectively uses to improve its performance.

**TABLE 13:** Performance of the CNN-Bi-LSTM for Classifying Whether a Virus Gene Contains Regulatory Motifs for the SARS CoV-2 Genes or Not

	Sample size (%)	CNN-Bi-LSTM
Sens(%)	25	99.76
	50	99.79
	75	99.99
	100	99.76
Spec(%)	25	99.76
	50	99.79
	75	99.99
	100	99.76
Prec(%)	25	99.99
	50	99.79
	75	99.99
	100	99.76
Acc(%)	25	99.71
	50	99.98
	75	99.99
	100	99.98
AUC ROC(%)	25	98.9
	50	99.85
	75	100.00
	100	100.00
MCC	25	0.998
	50	0.998
	75	0.999
	100	0.998
Cohen’s Kappa	25	0.997
	50	0.998
	75	0.999
	100	0.997

### C. Identifying Nucleotides in Regulatory Motifs for the SARS CoV-2 Genes Using Saliency Maps

In this paper, we use the saliency map to show which bases in a virus gene sequence are important for predicting whether the sequence contains regulatory motifs for the SARS CoV-2 virus gene or not. Moreover, the map shows the gradient of the model’s prediction for each nucleotide. This means that the saliency map shows the changes in the output response value (i.e., whether a sequence contains regulatory motifs or not) concerning small changes in the input nucleotide sequence [20]. The gradients can be positive or negative and all the positive values in the gradients tell us that a small change to that nucleotide will change the output value.

**FIGURE 4.**
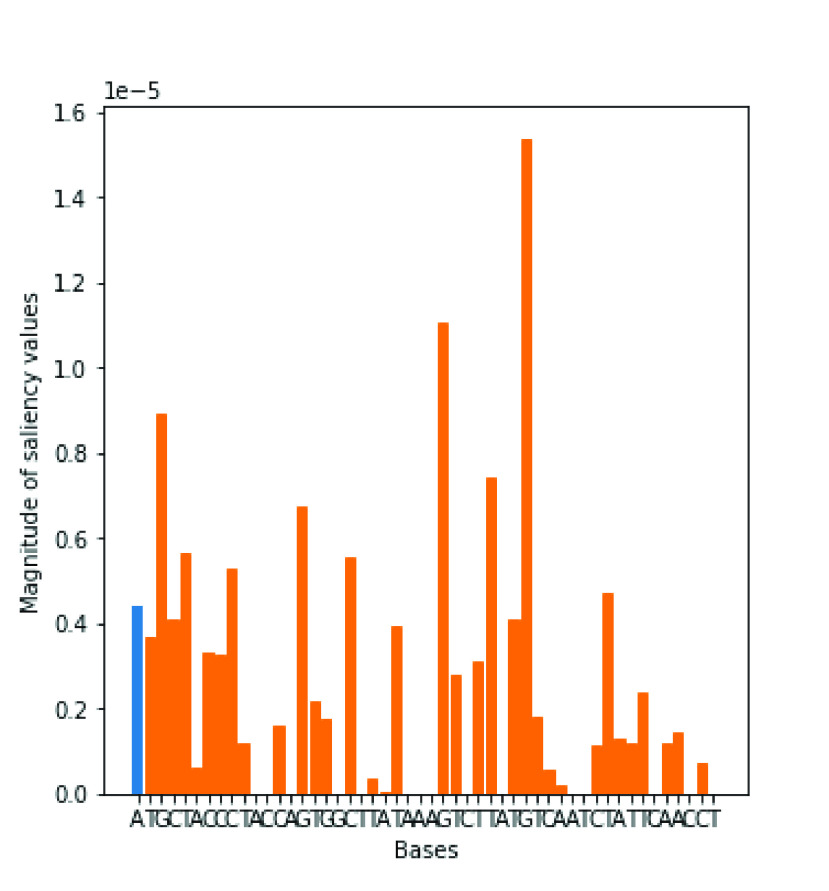
Saliency map for bases in one of the positive samples (orange indicates the actual bases in motif).

Using our best performing model (CNN-Bi-LSTM model), the saliency map shown in Fig. 4 shows the bases that have high magnitudes of saliency values. Bases with high saliency values are important for predicting the sequence contains regulatory motifs for the SARS CoV-2 virus or not. The saliency map has therefore revealed nucleotides that are responsible for predicting whether a virus gene has regulatory motifs for the SARS CoV-2 virus gene.

## VII. Discussion

The main findings from the performance evaluations of the deep learning models are: 1) at peak, the CNN-Bi-LSTM achieves performance scores for Sens, Spec, Prec, Acc, AUC ROC that are comparable to those of the CNN and CNN-LSTM models; 2) the CNN-Bi-LSTM, CNN-LSTM and CNN models produced high scores on the more reliable statistical measures, the MCC and Cohen’s Kappa, which are used to measure the quality of binary (two-class) classifications. The high MCC and Cohen’s Kappa values show that all these models are useful for binary classification, an indication that the models obtained excellent results in all of the four confusion matrix categories (true positives, false-negatives, true negatives, and false positives); 3) our proposed model, the CNN-Bi-LSTM can classify the SARS CoV-2 virus, which is very similar to other viruses in the Coronaviridae family; 4) the $5 \times 2$-fold cv paired ${t}$-tests shows that at peak, the CNN-Bi-LSTM achieves an AUC ROC of 100% which is significantly higher than that of the CNN and CNN-LSTM models. Consequently, the proposed CNN-Bi-LSTM model achieves good binary classification results; 5) the ${P}$-value [Acc > NIR] for CNN-Bi-LSTM (2.2e-16 < 0.05), CNN-LSTM (2.2e-16 < 0.05) and CNN ($\text {2.2e-16} < 0.05$) were all significant at a 5% level of significance. These results show that the classification accuracy is significantly greater (at 5% level of significance) than the NIR. This means that the deep learning models are useful for predicting 1s (positive samples) and 0s (negative samples) even when using unbalanced data. We used the ${P}$-value [Acc > NIR] because the accuracy may not be sufficient as a measure of performance especially in our case where the datasets are imbalanced.

The primary goal of this paper was to develop a classifier (CNN-Bi-LSTM) that could efficiently distinguish between SARS-CoV-2 gene sequences from non-SARS CoV-2 gene sequences and then compare its classification performance to that of the CNN and CNN-LSTM classifiers. Based on experimental results and the $5 \times 2$-fold cv paired ${t}$-test, the CNN-Bi-LSTM outperformed the CNN-LSTM and CNN models in classifying SARS CoV-2 gene sequences relative to the AUC ROC. The AUC ROC is a better measure for differentiating between classes. For example, if AUC $\text {ROC} = 1$, then a classifier is able to perfectly distinguish between all the SARS CoV-2 gene sequences and non-SARS CoV-2 gene sequences. The differences in performance between the CNN-Bi-LSTM and the other models is statistically significant at 5% level of significance as shown by the $5 \times 2$-fold cv paired ${t}$-tests in Tables 8 and 9. This shows that the CNN-Bi-LSTM model can be used as an alternative model to the CNN and CNN-LSTM. The CNN-Bi-LSTM model takes advantage of the ability of the CNN layers to extract as many features as possible from the DNA sequences. Besides, the model uses the Bi-LSTM layers to learn past and future states in making predictions as well as using the temporal features present in DNA sequences. The Bi-LSTM can keep the chronological order between data, which is very important when analyzing long DNA sequences. Thus, by combining these two models into a CNN-Bi-LSTM, we have created a model that takes advantage of the power of the CNN in capturing features that are then used as the input for the Bi-LSTM layers. Therefore, we have developed a hybrid model that meets the objective of efficiently classifying SARS-CoV-2 among Coronaviruses. The CNN-Bi-LTSM model consists of three convolutional layers followed by max-pooling layers and a single Bi-LSTM layer as well as a fully connected dense layer fully connected neural network layer which contains 100 neurons for classification. The convolutional layers had 32 kernels and the Bi-LSTM had 32 cells. The results of Tables 4 and 5 show that increasing further the number of kernels in the CNN and the number of cells in the Bi-LSTM was not beneficial as there were no significant improvements in the performance of the proposed model. Based on the findings by [19], we used three convolutional layers because using additional layers of convolution and max-pooling may make the neural network harder to train because it is now “deeper”. Reference [22] utilised two convolutional layers followed by max-pooling when classifying DNA sequences using the CNN model. Table 6 shows that the training time for 100 epochs also increases with model complexity, the CNN-Bi-LSTM has an additional bi-directional layer that uses information from past and future states simultaneously, thus, it can understand the context better. Also, Table 6 shows that the overall number of parameters for the CNN model is greater than that of the CNN-Bi-LSTM and CNN-LSTM models. The CNN model contains 31 394 trainable parameters, and the CNN-Bi-LSTM contains 27 892 trainable parameters. The CNN has 12.56% more parameters. This difference in the number of trainable parameters is a result of differences in the size of the dense layer of the two models. The dense layer of CNN models is connected to all the values of the preceding layer and will require a larger weight matrix to parametrise the connection. Conversely, the feature map is processed sample by sample by the CNN-Bi-LSTM model using the recurrent Bi-LSTM part of the model. Therefore, the CNN-Bi-LSTM will require a much-reduced number of parameter values. We note that even though the CNN-Bi-LSTM is a complex model compared to the CNN model, it has fewer parameters. This has implications on the computational resources required when using the CNN-Bi-LSTM model.

We included in the CNN part of the model 1D max-pooling layers but in practice, this is not always the case as reported by [31]. We used the max-pooling layers to reduce the number of parameters that the models need to learn and thus reduce the training time required. Therefore, the max-pooling layer performs a down-sampling of sequential data via the 1D max-pooling operation. In this paper, we focused more on optimising hyperparameters that influence the network architectures such as the number of kernels in CNN layers as well as the number of cells in the LSTM layers, that have an impact on performance. We observed that those parameters such as the learning rate and the dropout technique had less effect on performance. For example, we used drop-out rates equal to 0.1, 0.3, and 0.5 yielding little difference in terms of performance. Also, this finding is supported by [67], [68].

Additionally, we demonstrated that our proposed model was robust enough when applied to new data (datasets for classifying whether a gene sequence contains regulatory motifs for the SARS CoV-2). Table 13 shows the performance of the CNN-BILSTM model when applied to datasets of increasing cardinality. As the cardinality of the datasets increased, there were no significant improvements in performance. This shows the robustness of our proposed model as it is capable of obtaining a very good performance even with relatively small datasets. This finding seems to indicate that although deep learning techniques are often employed with large amounts of data, they may be applied in situations where obtaining large and labeled datasets may be costly.

## VIII. Limitations of the Study and Future Work

Deep learning models require more time to train. This is because they have a large number of parameters that need to be trained. Well-trained models are often computationally demanding and they also require large memory. Thus, the deployment of deep learning models can be hampered by computational and memory requirements in cases where there is limited computational power. Thus, in this paper, we could not develop “deeper” architectures as they require more computational resources. Another limitation of our deep learning approach is that the models do not offer easily available explanations on how SARS CoV-2 gene sequences are classified in a particular way, compared to the alignment-based methods. Thus, we used deep learning models more as “black boxes” without providing an explainable justification for their classification results. Additionally, our deep learning models require a large set of training data, as opposed to alignment-based methods that can work even with one reference genome sequence per class. Thus, deep learning models require several examples per training class. Despite these limitations, the deep learning methods were able to correctly classify SARS Cov-2 amongst Coronaviruses and also classify whether a sequence contains regulatory motifs for the SARS CoV-2 or not.

For future work, we may evaluate the effect of increasing the number of both convolutional and Bi-LSTM layers subject to the availability of computational resources to find a trade-off between how a model performs versus training time. Still, for future work, we will also recommend investigating the causal effect of changes in the composition of the regulatory motifs. Besides, we recommend the use of our proposed model to classify other viral genes as well as explore RNA-protein binding predictions.

## IX. Conclusion

When there is a viral disease outbreak such as that of COVID-19, there is a need for an understanding of the virus’s genomic sequence to swiftly act towards containing the virus, treating those that are affected by the virus, and developing vaccines that help to disrupt the spread of the virus. Current tools that are used to detect the virus such as the molecular technique and RT-PCR require support from newer and faster deep learning methods. Thus, it is vital to develop diagnostic tools capable of reliably identifying the SARS CoV-2 virus and then distinguishing it from other Coronaviruses or pathogens. These newer methods help in improving the detection rate. Since the SARS CoV-2 is very similar to other Coronaviruses, the other Coronaviruses can exhibit respiratory infections that are the same as those of SARS CoV-2. Consequently, the identification of the SARS CoV-2 becomes a challenge. It is, therefore, essential to carry out similarity comparisons that can timeously differentiate a novel virus such as SARS CoV-2 from other viruses that are comparable. The similarity comparisons of the SARS CoV-2 virus with other similar and known viruses are crucial in distinguishing whether a DNA sequence is that of SARS-CoV-2 or not. Traditionally, alignment-based methods such as BLAST can be time-consuming. These methods can face challenges when comparing large numbers of sequences that have significant differences in their composition. The advantages of using alignment-free approaches are that they have a quick turn-around in producing desired results and they can simultaneously handle a substantial number of sequences at the same time.

In this paper, we were able to easily compare short sequences of genes with different compositions that were coming from different regions of a complete genome sequence. For example, the **orf1ab** virus gene from SARS CoV-2 was labeled as a negative sample even though it came from the same sequence (SARS CoV-2 virus complete genome sequence) as other positive sequences that came from the same SARS CoV-2 gene sequence.

We combined a CNN and Bi-LSTM to classify SARS CoV-2 genes from other Coronaviruses as well as classify whether a genome sequence contains regulatory motifs that serve as binding sites of transcription factors that regulate the expression of SARS CoV-2 genes. Besides, correct classification is important in discovering different species of Coronaviruses, which may affect people in the future. Besides, the SARS CoV-2 virus gene is highly transmissible, hence the proper identification of the SARS CoV-2 is very important in the management of the spread of the virus. Our experimental results using the SARS CoV-2 datasets have shown that the CNN-Bi-LSTM has outperformed the CNN and CNN-LSTM and it can be applied to identify accurately SARS CoV-2 gene virus amongst Coronaviruses. The CNN-Bi-LSTM can effectively and efficiently classify DNA sequences datasets of varying cardinalities that it had not seen before. Our proposed model, the CNN-Bi-LSTM outperformed the CNN and CNN-LSTM in detecting whether a virus gene contains regulatory motifs for the SARS CoV-2 virus. Using saliency maps we were able to identify the nucleotides or bases that are important in predicting whether a given gene sequence contains regulatory motifs for the SARS CoV-2 or not. By identifying candidate regulatory motifs together with the bases that predict whether a given sequence is that of SARS CoV-2 or not, it enables scientists to understand the virus’s regulation mechanism(s) of gene expression.
